# Integrating Older Adults’ Voices on Climate change Into One Health Framework to Promote Healthy Aging

**DOI:** 10.1093/geroni/igaf122.3968

**Published:** 2025-12-31

**Authors:** Hui Zhao, Anne Stewart, Francesca Ezeokonkwo, Joey Oi Yee Wong, Kayoung Lee, Karen Lok, Yi Wong, Kai Zhang, Lillian Hung

**Affiliations:** James Madison University, Harrisonburg, Virginia, United States; James Madison University, JMU Dept of Graduate Psychology, Virginia, United States; Holy Family University, Philadelphia, Pennsylvania, United States; University of British Columbia, Vancouver, British Columbia, Canada; University of British Columbia, Vancouver, British Columbia, Canada; University of British Columbia, Vancouver, British Columbia, Canada; UNT Health, Fort Worth, Texas, United States; University of British Columbia, Vancouver, British Columbia, Canada

## Abstract

Climate change poses threats to population health, affecting not only physical health, but also mental, social, and environmental well-being. Older adults are especially vulnerable, yet their perspectives are often overlooked in climate and health research. This qualitative pilot study foregrounds the voices of older adults, applying the One Health Framework to examine their perspectives on how climate change affects overall health. Employing an autophotography process and semi-structured interviews, the study also explores older adults’ knowledge and beliefs about climate-related issues. Seventeen community-based older adults were recruited and 14 of them completed the study. Participants are white, aged 67-85, and mostly with graduate degrees. Constant comparison was used to analyze data. Key themes included 1) Vulnerability—participants highlighted challenges such as self-care and inadequate infrastructure during climate change and linked these challenges to their impacts on participants’ care for plants and wildlife; 2) Recognizing Ecosystem Disruption—participants noted disruption of local weather patterns, and food systems and changing patterns in plant and wildlife interactions; and 3) Nurturing Resilience—participants described adapting strategies, including engaging in energy saving, plant care and sustaining strong bonds with wildlife as sources of well-being. Participants emphasized the need for cross-sector collaboration and intergenerational knowledge sharing to strengthen resilience. Findings indicate that acknowledging older adults’ perspectives is critical for achieving holistic health goals. Within the One Health Framework, these insights highlight the interconnectedness of human, wildlife, and environmental health, and confirm that older adults’ health outcomes cannot be addressed in isolation from ecological and social systems.

